# Applying EMG technology in medial and lateral elbow enthesopathy treatment using Myo motion controller

**DOI:** 10.1007/s13246-019-00770-5

**Published:** 2019-06-14

**Authors:** Adam Grabczyński, Krzysztof Szklanny, Piotr Wrzeciono

**Affiliations:** 1 Multimedia Department, Polish-Japanese Academy of Information Technology, Warsaw, Poland; 2 Faculty of Applied Informatics and Mathematics, Warsaw University of Life Sciences-SGGW, Warsaw, Poland

**Keywords:** Myo movement controller, Electromyography, Tennis elbow, Rehabilitation

## Abstract

Electromyography (EMG) is a diagnostic technique allowing for the detection of signals generated by changes in electrical potentials of striated muscles. The application of this technology is becoming an increasingly popular subject of scientific research. With the appearance of new devices retrieving EMG data, novel methods of its processing for various purposes are being developed. One such device is the Myo movement controller, produced by Thalmic Labs (now North). The device has been used for the analysis of muscle activation levels in patients with "tennis elbow" and "golfer’s elbow"—conditions of upper limbs which usually result from occupational injuries. The process of their rehabilitation is complex and requires a continuous monitoring of its progress. The data obtained by means of the Myo controller was used for pattern recognition of an injured hand with relation to the healthy one. The study involved examining ten subjects, including five controls. The results indicate that the muscle activation force is considerably lower in injured individuals. The arithmetic mean for the 6 analyzed motions in the injured group is 38.54% lower. The SmartEMG application (https://www.smartemg.com) enables the implementation of procedures performed during an examination as well as those involved in the management of the collected recordings. The study produced satisfactory results, which indicates the possibility of using the Myo controller in the treatment of elbow enthesopathy.

## Introduction

Electromyography (EMG) is a concept related to electrophysiology, an area encompassing the electrical activities in the body. It is a method of signal detection based on the changes in electrical potentials of striated muscles. Electromyographic readings are not easy to interpret, because they are influenced by many factors. The quality of the used equipment and the high complexity of muscles in the body hinder unambiguous determination of where the registered signal comes from. Apparatus consisting of cables connecting electrodes with the signal amplifier restricts the freedom of movement, impeding the research [1]. Surface electromyography, which is more commonly and more frequently applied, is free of these defects. Electrodes placed on the skin surface carry no risk of infection, do not cause pain or unpleasant adverse effects, and do not require medical supervision [2]. However, they provide unspecific data which is a sum total of signals from many sources, generated by different motoric units which are located within the electrode sensitivity range. It is also difficult to determine which muscle group sends which signal, which is a definite drawback. To interpret such results more accurately, sets of multiple surface electrodes are used together with advanced algorithms of signal processing and pattern recognition. There are many professional devices which were developed for specific scientific studies, e.g. those concerning electromyographically controlled prostheses [3] or the development of a system for classification of gestures with the use of a 3D accelerometer [4]. Also, a small portable wireless prototype of a system using EMG electrodes was developed for the electrostimulation therapy of patients with motor paralysis [5]. Electromyographies are used in the case of paresis following a stroke [1], for electrostimulation of muscles in patients suffering from hemiplegia [5] and in many other cases [6–13].

One of those advanced systems is Noraxon Myosystem 1400 L [6]. It is an example of a precision apparatus that is fully wired, which limits the possibilities of research mainly to static measurements. There are also some expensive wireless sensors which allow a greater freedom of movement [14], and portable data recorders.

In 2014, Thalmic Labs (now North), a Canadian start-up, launched the Myo movement controller. The Myo movement controller [15] differs from the above-mentioned devices mainly in that it is not medical equipment.

The Myo movement controller is an armband, 48 mm in width and 11 mm in thickness (at its thickest point), which weighs 93 g and is available in black and white colors. Myo consists of eight elements: three larger parts and five smaller ones. They are connected by a rubber frame which extends while the whole device is attached to the forearm. The three notably larger segments contain computing units and an energy source. The device is powered by two lithium-ion batteries with a capacity of 260 mAh each, which enables continuous operation for a whole day. The device operates with an ARM Cortex M4 processor. Each of the 8 elements is an electromyographic signals sensor containing 3 metal electrode contacts, and a signal amplifier. The device also features a 9-axis IMU unit, which is a module measuring spatial movements, containing a 3-axis gyroscope, 3-axis accelerometer and 3-axis magnetometer. Thus, the controller uses two sources of information: EMG sent with a frequency of 200 Hz and IMU sent with a frequency of 50 Hz [15, 16]. Feedback is provided to the user by a vibration engine with different levels of vibrations. The operation of the controller is based on the interpretation of spatial hand gestures and arm movements. Myo by default recognizes only 5 user gestures (fist, fingers spread, wave in, wave out and double tap). The device has been the subject of numerous works [15, 17–20]. Its production and sale officially ended as of Oct 12, 2018, although Myo customers are still supported.

The aim of this study was to compare the normalized muscle activation force in patients with tennis elbow and golfer’s elbow with the muscle activation force in healthy individuals. The study involved an analysis of fist grip, palm hyperextension with resistance, palm flexion with resistance, pressing the hand against the occiput, pressing the hand against the sternum, fingers spread with resistance.

The treatment of these injuries is a complex process and requires a continuous monitoring of the rehabilitation progress and the effects of invasive procedures. The development of a tool to help determine the condition of the patient by recording the results in a digital form may facilitate the process of supervision and assessment of the effectiveness of the course of treatment.

## Tennis elbow

“Tennis elbow” is one of the most common injuries of the forearm [21]. It is diagnosed most often in persons between 36 and 65 years of age, regardless of sex, and it affects 1.3% of the general population [22]. It is estimated that 40–50% of all recreational tennis players will suffer from tennis elbow in their lives [23]. However, the problem is not restricted only to tennis players. The following factors contribute directly to the injury: work with heavy tools, activities such as the manual fixing of screws, moving heavy objects and movements involving elbow and wrist extension [22]. The cost of surgical treatment of tennis elbow (in the USA) is 9.8 times higher than the cost of treatment without surgical procedures and amounts to approximately 6000 USD [24].

Patients may complain about pain in the area of lateral epicondyle of the humerus while holding objects, or about a weakened grip strength. Hypersensitivity of this region occurs along the wrist extensor. The results include decreased strength, longer electromechanical reaction time, and a decreased RFD index, which determines the maximum body strength in a time unit, i.e. the pace of strength development. In dynamometric testing, patients with a diagnosed tennis elbow present notably lower free-from-pain grip strength in the position of extended elbow than those with no such injury [25]. The diagnosis is based on tests involving the lifting of a chair, mug or bottle in positions that engage the work of the wrist extensor against assigned resistance, and also the extension of the middle finger with resistance [23].

Depending on the degree of advancement of enthesopathy of the lateral epicondyle of the humerus, different methods of treatment are applied. In 90% of all cases, non-surgical treatment proves to be effective. It consists of physiotherapy, administration of anti-inflammatory medication, corticosteroid injections, or platelet-rich plasma injections. Physiotherapy includes the stretching of wrist extensors, the improvement of blood flow in tissues, eccentric exercises therapy (exercises in which muscles work more during extension than during flexion) [26, 27], stabilizing the elbow part and wrist, and various methods of physical therapy [23, 28]. Corticosteroid injections are a solution that brings short-term relief. Surgical procedures are used when no improvement occurs for 6–12 months despite the application of different techniques of non-invasive treatment or minimally invasive treatment.

## Golfer’s elbow

“Golfer’s elbow” causes pain where wrist flexors attach to the medial epicondyle of the humerus. The pathogenesis of this injury is similar to enthesopathy in the case of “tennis elbow,” but it concerns the wrist flexors and other flexor muscles in the forearm. Inflammation and tissue degeneration are also present. Pain occurs during resisted wrist flexion, finger flexion, e.g. when shaking hands, or forearm pronation.

This injury occurs much less frequently than tennis elbow, with approximately 0.4% of the general population being affected [21]. It occurs most commonly in men in the dominant hand [29]. It is caused by excessive muscle strain while performing activities against resistance, particularly when the activity is of a monotype nature, e.g. working with a monkey wrench, screwdriver, hammer, or weight lifting. While the activities are similar to those that lead to tennis elbow, here the overload of the tendons of the medial epicondyle is largely caused by the flexion movements of the wrist against resistance. Both types of enthesopathy of the humeral epicondyle are common among meat cutters, chefs and machine industry workers. Athletes who throw objects with particular force exploit their flexor muscles while performing a throw. For baseball players it is the medial epicondyle itself that is most prone to injury [23]. However, as with tennis elbow, the etiology of the injury very often lies in recreational activities. The manner of diagnosing this injury is similar to that of tennis elbow and so is the treatment.

## Methods

### Characteristics and processing of EMG signal

Muscles are mainly responsible for movement, posture and heat generation. They can be divided into three types: smooth muscles, which are involuntary and are responsible for actions such as bowel movements, cardiac muscle (unique in its structure) and skeletal muscles. The latter are striated muscles, responsible for the motility of the skeleton, including posture and limb movements. Muscles receive signals sent from the brain through the nervous system. These signals are transmitted via the spinal cord right to the executive unit. Each muscle is innervated by a single motor neuron. A signal from a single motor neuron causes the muscle fibers connected with it to contract, forming a coherent, integrated motor structure [1, 19, 20]. This signal triggers a sequence of electrical and chemical reactions leading to the polarization and depolarization of muscle fibers. As a result, an electromagnetic field, or the so-called action potential, is created in each fiber, which can be detected using electromyographic equipment. A surface EMG electrode receives an aggregated signal of action potentials coming from many various muscles that occur in the range of its sensitivity. The reading is then a complex electromagnetic field in a given time unit [1, 20]. The frequency of the detected EMG signal is usually within the range of several to several hundred Hertz and before the amplification reaches the amplitude within the range of zero volts to millivolts. This signal is then amplified even several hundred times, which is why it is essential to obtain good signal quality before amplification with the least possible level of noise. The obtained EMG data may be presented in the form of an amplitude graph [mV] depending on time [s], which makes it possible to analyze aggregated muscle activation values in time. It is also possible to present such data in the form of a graph showing the interrelation between muscle activation impulses and frequency [Hz]. This allows one to detect the number of muscle activation impulses that occur for the respective signal frequencies. Consequently, we can determine which muscle groups are working harder, knowing that some muscles are activated at lower frequencies, while others—at higher ones [20, 30].

### SmartEMG

To recognize patterns characteristic of golfer’s and tennis elbow, a web application—SmartEMG—was developed. The application was written using open-source technologies commonly referred to as “MEAN”, i.e. MongoDB, Express.js, AngularJS and Node.js. These technologies create an environment in which JavaScript is the main application language, both on the client and server side. MongoDB is a NoSQL type database which stores documents in a JSON type format. The graphic composition of the web application SmartEMG together with its functionality is developed in a user-friendly manner, in compliance with good practice of UX Design and affordance, which allows the recipient to understand the function of a given element with ease [31]. The dark theme of the portal is aimed at reducing eye strain, which can be caused by the commonly used bright themes. The structure of the whole portal is responsive, which means it displays correctly on mobile devices. SmartEMG (https://www.smartemg.com/) enables connection to Myo Connect through WebSocket, as well as the recording and management of data. The application supports the registration of multiple users, creation of patient database and the development of patient health check history. The portal was developed to create a physician-friendly tool. It has a simple and clear design and can be operated by those who are not necessarily computer-savvy. Checks may be performed concurrently by many physicians on different patients, and sensitive operations (such as the deletion of patients) can be performed only with relevant authorizations. Upon logging in to the SmartEMG system, the user can access data from all the Myo controller sensors in real time and record them while performing diagnostic movements. During the recording process it is possible to mark the moment at which the pain occurs. Movements and patients are added separately in a separate system section. Recorded data may then be viewed in a relevant system section or downloaded through API.

### EMG data processing

Three methods of computing the maximum values of muscle activation force were prepared. The first one consists in computation the sums of waveform lengths [3]. This method is expressed with the formula below (1), where x(k) indicates a given sample, x(k − 1) is a previous sample and N is a number of all samples in a given window:

1 $$EMG_{WL} = \mathop \sum \limits_{k = 2}^{N} \left| {x\left( k \right) - x\left( {k - 1} \right)} \right|$$

For the given wavelength window size, the script computes the signal for each of the eight sensors in a given movement. The obtained wavelength sum contains the processed record of all eight sensors. The sliding window moves with each sample and computes RMS over the data in every iteration. The next method used is Root Mean Square (RMS), expressed by the formula below (2), where x(k) is a given sample and N is the number of samples in a given window:

2 $$EMG_{RMS} = \sqrt {\frac{1}{N}\mathop \sum \limits_{k = 1}^{N} x\left( k \right)^{2} }$$

For the application of formula (2), the dsp.MovingRMS() function available in Matlab was used. The step() function processes raw EMG data with a set method and returns the data with an approximation of muscle activation strength. The third method of processing is a combination of the first two methods described above. The raw signal is first processed with the WL method (1), and then with the RMS method (2). In this case the step function is activated on wavelengthSum data processed earlier with the WL method. Upon obtaining the results of all three methods, the sensor with the highest strength value is determined, and its maximum value is established. The operation is repeated for each of the methods.

Differences are computed based on all the gathered, processed and structured data. The WavelengthSumMax (WL) and wavelength SumRMSMax (WL and RMS) values exceed the scale of 0–128 for rawRMSMax. This is the consequence of applying the WL method, which consists in the summing up of samples in a given window. These values are used for the computation of the ratio of one hand versus the other, so the change of scale makes no difference here. Based on such data structure, the difference between the activation strength of both hands is calculated. The main and final determinant of the analysis is the ratio of the dominant hand values to the values of the non-dominant hand of the patient.

## Results

The research was carried out in a group of ten individuals referred to as “patients” in the portal, five of whom suffer from golfer’s elbow and/or tennis elbow, and the remaining five constitute a control group with no injury (4 males and 1 female). An orthopedist examined the patients with a diagnosed enthesopathy of the elbow. Only the patients with enthesopathy in one hand were selected. Table 1 presents information on the disease of the patients. Among five patients there was one person (P3) whose injured hand is non-dominant, which is a less frequent case [25, 27, 29].

**Table 1:** Patient data

Patient ID	P1	P2	P3	P4	P5
Diagnosis	Golfer’s elbow	Golfer’s and tennis elbow	Tennis elbow	Tennis elbow	Tennis elbow
Treatment	None	None	During rehabilitation	None	After corticosteroid injection
Sex	Male	Male	Female	Male	Female
Pain in the hand	Right	Right	Left	Right	Left
Dominant hand	Right	Right	Right	Right	Left

The study consisted in recording a motor activity of each patient using the Myo in SmartEMG portal. Each examined person performed 6 motions with their left hand and 6 motions of the same type with their right hand. The description of motions in question is presented in Table 2. Each of the indicated motions was performed with a maximum strength, within the pain-free range. In the case of an injured arm, the examined person performed the motions with a gradually increasing intensity until reaching the pain threshold. To facilitate the detection of this moment, the examined person had performed a prior non-registered testing motion. Between each motion, a minimum of 30 s break was maintained to allow the muscles to regenerate.

**Table 2:** Motions taken into consideration in research

Fist grip	Movement of clenched fist in an intermediate position
Palm hyperextension with resistance	Movement of palm hyperextension of the hand in intermediate position, without clenching fingers, with resistance in the hand of the examiner
Palm flexion with resistance	Movement of palm extension of the hand in intermediate position, without clenching fingers, with resistance in the hand of the examiner
Pressuring the hand onto the occiput	Pressuring with fingers of the extended hand to one’s occiput with elbow waved out to the side
Pressuring the hand onto the sternum	Pressuring with fingers of the extended hand just over one’s sternum, with elbow waved out to the side
Fingers spread with resistance	Extension of fingers with arranging them one to another, and with applied resistance

## Discussion

The EMG signal from the Myo controller consists of samples measured with a frequency of 200 Hz and reaching the values within the range from − 128 to 127. The raw EMG data was processed with the help of three methods: the RMS, the sum of wavelength (WL), and a method that combines the first two (WL-RMS). Then, the ratio of the activation strength of the dominant limb muscle was assessed in relation to the non-dominant one. This helps to determine the proportion of the strength of both hands in healthy people and those with enthesopathy of the lateral and/or medial epicondyle.

**Fig. 1:**
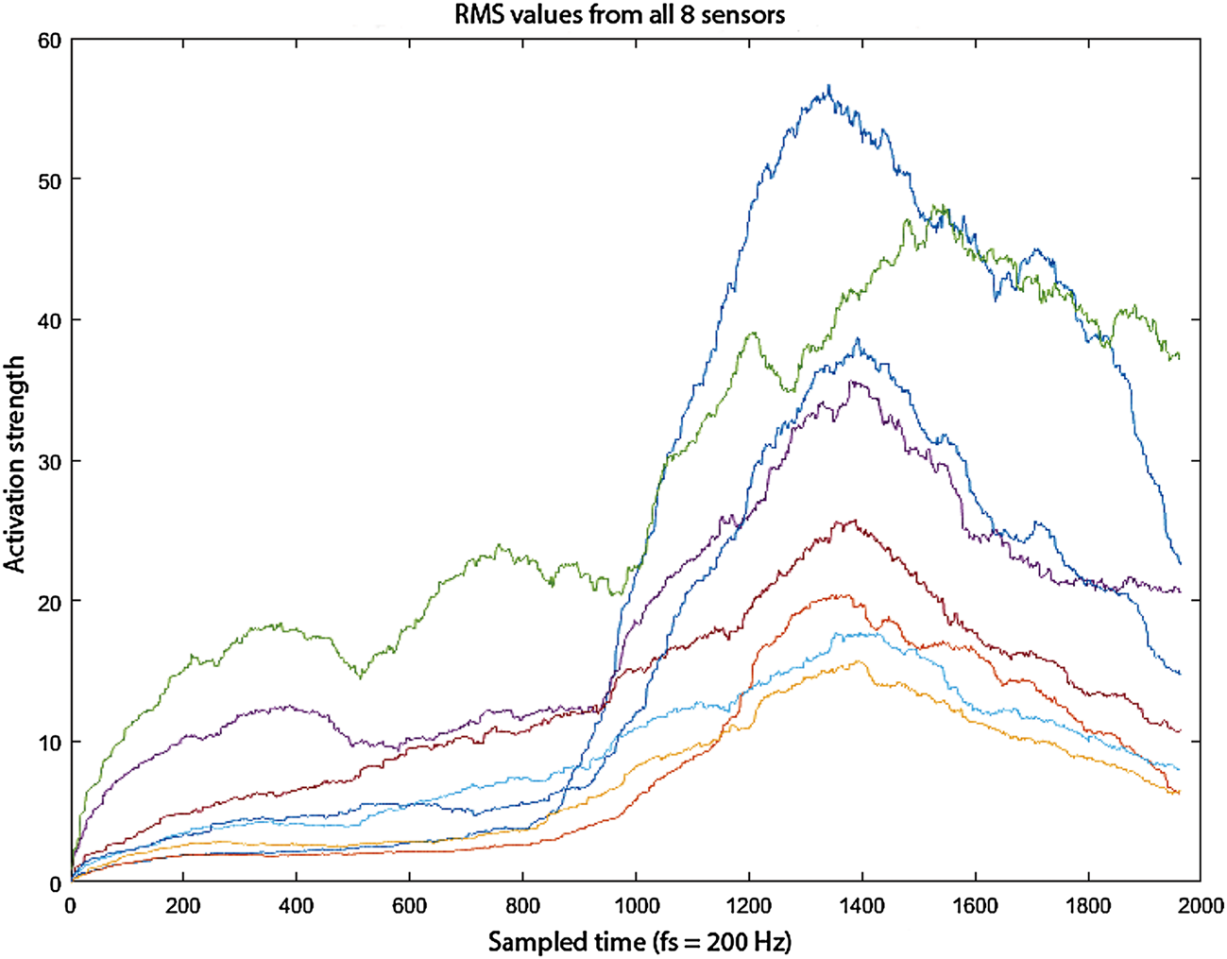
RMS approximation of activation strength for each of eight sensors

Figure 1 presents EMG data processed with the RMS method for each sensor. As we can see, some of the sensors show significantly higher activity than the others. This results from a stronger operation of muscles in a given part of the forearm. Thus, it is possible to locate the part of the forearm with the biggest activation strength. In this study, the sensor with the strongest signal for each movement of both hands is selected.

**Fig. 2:**
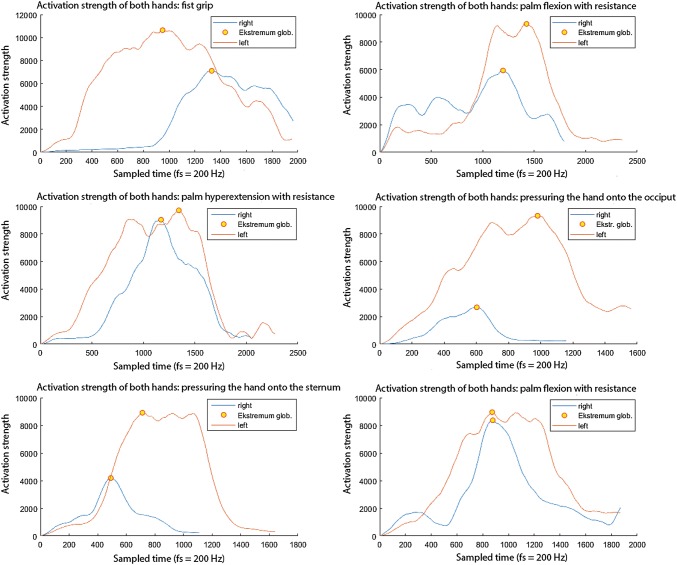
Overview of all P1 patient movements, WL-RMS method

The correlations between the described methods are shown in Fig. 2. The different window sizes (100 and 200 samples) were tested. The bigger the size of the window, the smoother the function, though that at the cost of losing the particulars of local extremes. A too big window also suppresses the global extreme when it is located at the beginning or at the end of the recording, when the movement has not yet been performed but the recording is already on. All three methods clearly display the outline of the muscle activation force. The RMS method with a 200-sample window presents an accurate level of detail and approximation. A stronger emphasis on abrupt changes characterizes the WL method with a 100-sample window. The WL-RMS method, with 100-sample windows, seems to combine some of the advantages of the other two methods. It also gives the smoothest graph while still clearly reflecting the variability of the signal. However, just like in WL, the scale without amplitude unit values changes.

By grouping the movements into left–right pairs, it is possible to observe their activation force. Figure 2 shows the results for patient P1 (Table 1) processed by the WL-RMS method. This patient’s injury is located in his dominant right hand. It is visibly weaker than his non-dominant left hand. It can also be seen that some of the movements are significantly different from others. In these movements the wrist flexors are more active and the injured hand produces worse results. It can then be concluded that the patient suffers from golfer’s elbow, which is compliant with the relevant medical diagnosis.

## Comparison of the strength of both hands

What poses a significant problem in the processing of EMG data is the normalization of signals. The solution to this problem consists in comparing the results of the patient’s injured hand to his healthy hand, which serves as a reference point. To determine whether the ratio of both hands deviates from the standard, it was checked what proportions occur in healthy people. For this purpose, a control group was examined, their results being presented in Table 3. These are percentage values expressing the dominant to non-dominant hand ratio. The values in this table are arithmetic means of the results from all three methods. The arithmetic mean of the strength of all movements for WL RMS equals 107.23%, which means that on average the dominant hand in healthy people is stronger than the non-dominant hand, and this is the percentage of its activation force. The average for all methods equals 107.46%.

**Table 3:** Comparison of methods based on average results for the control group

Movements of healthy subjects	RMS (%)	WL (%)	WL RMS (%)	Arithm. mean (%)
Fist grip	100.35	92.29	92.50	95.05
Palm hyperextension with resistance	104.41	109.27	109.10	107.59
Palm flexion with resistance	117.07	116.17	117.31	116.85
Pressuring the hand onto the occiput	109.91	107.19	106.64	107.91
Pressuring the hand onto the sternum	112.69	114.71	115.43	114.27
Fingers spread with resistance	105.24	101.59	102.41	103.08
Arithm.mean	108.28	106.87	107.23	107.46

The same calculations were performed on the group of injured patients. Table 4 presents the average dominant to non-dominant hand ratio for each patient. In Table 4, the case of patient P3 is distinctive, because its values significantly exceed 100%. Patient P3 suffers from enthesopathy in his left hand, but his right hand is the dominant one, which is a less frequent case.

**Table 4:** Results for injured patients, the WL-RMS method

Patient movements	P1 (%)	P2 (%)	P3 (%)	P4 (%)	P5 (%)	Movement arithm. means (%)
Fist grip	66.71	92.29	96.64	101.88	88.94	89.29
Palm hyperextension with resistance	63.72	85.22	85.51	47.09	77.82	71.87
Palm flexion with resistance	92.98	48.08	61.63	39.95	87.87	66.10
Pressuring the hand onto the sternum	28.68	61.96	44.06	61.38	86.35	56.48
Pressuring the hand onto the sternum	47.03	37.05	67.37	42.78	79.59	54.76
Fingers spread with resistance	93.42	45.99	71.86	67.70	78.42	71.48
Average patient result	65.42	61.77	71.18	60.13	83.16	

Arithm. mean of the activation strength of the dominant hand 68.33% versus non-dominant

This means that the dominant to non-dominant hand ratio increases. Thus the result confirms the medical diagnosis of P3 patient. In the remaining patients the results also match the expectations. For P3 patient it is possible to calculate the value proportionate to the other patients.

In this case the method of calculation is expressed in formula (3), where *x* means the percentage determinant for the patient with a non-dominant hand injury, *a* means the average percentage value of the dominant-non-dominant hand ratio in healthy people (Table 3), and *p*_3_ is the already computed ratio of the dominant healthy hand to the non-dominant injured hand:

3 $$x = \frac{a}{{p_{3} }}$$

The x value then describes ratio in which the injured hand is weaker with reference to the average, healthy hand strength ratio.

Table 4 contains results for patients and a column for patient P3 computed according to the formula given above. P5 patient has the best results. This patient was examined two weeks after being administered a corticosteroid injection, which might have influenced these measurements.

C3 patient (marked) is a particular case of an injury in the non-dominant hand. A/P3 means the average of the ratio of healthy dominant to healthy non-dominant divided by P3 value.

Table 5 presents the results for patients and is analogous to Table 3 for the control group. The average activation strength amounts to 68.33%, which is compliant with the relevant medical diagnosis. The values significantly deviate from the previously set determinant 107.46% for a healthy pair of hands. At this stage, we can also see which motions in particular make the differences between the injured and the healthy hand most pronounced. The “pressing the hand against the occiput” motion as well as the “pressing the hand against the sternum” motion are clearly at the patients’ pain threshold. The advantage of these motions is the fact that when exerting resistance, the patient does not require the examiner’s assistance. In these motions, the examined person exerts resistance against his/her hand himself/herself with their own body.

**Table 5:** Results for all three methods, the WL-RMS method

Patient movements	RMS (%)	WL (%)	WL RMS (%)	Arithm.mean (%)
Fist grip	85.97	90.70	89.29	88.66
Palm hyperextension with resistance	74.81	72.50	71.87	73.06
Palm flexion with resistance	68.14	68.21	66.10	67.49
Pressuring the hand onto the occiput	60.11	56.85	56.48	57.81
Pressuring the hand onto the sternum	53.50	54.78	54.76	54.35
Fingers spread with resistance	72.38	72.59	71.48	72.15
Arithm. mean	69.15	69.27	68.33	68.92

With such data we can compare both studied groups. Table 6 presents the difference between the ratios obtained before. Muscle activation strength in injured individuals is clearly lower than in those without injury. The fist grip movement showed less visible differentiation between healthy and injured individuals in relation to the other motions.

**Table 6:** Difference between the results of control group and patients

Movements	Control group (%)	Patients (%)	Difference
Fist grip	95.05	88.66	6.39% points
Palm hyperextension with resistance	107.59	73.06	34.53% points
Palm flexion with resistance	116.85	67.49	49.36% points
Pressuring the hand onto the occiput	107.91	57.81	50.10% points
Pressuring the hand onto the sternum	114.27	54.35	59.92% points
Fingers spread with resistance	103.08	72.15	30.93% points
Arithm. mean	107.46	68.92	38.54% points

## Conclusions

SmartEMG enables the implementation of procedures performed during an examination as well as those involved in the management of the collected recordings. All of the users operated the portal efficiently, which means that the intended level of intuitiveness of the graphical interface was achieved. The Myo controller performed well with reference to its mobility. The ease with which the device can be carried, attached and taken off as well as its wireless Bluetooth connection are a great advantage, particularly in relation to other available devices that collect EMG surface data. The combination of Myo’s mobility with the multi-platform SmartEMG web-application significantly facilitated the research conducted in various locations. This also gives grounds to consider further development of this system with respect to telemedicine. Myo’s drawback is its lack of units of EMG signal amplitude. This may be a consequence of the complex data processing inside the controller itself. However, the data is characterized by surprising accuracy, considering the price of the device, its dimensions and the fact that no conducting gel is required.

The data processing functions in Matlab proved convenient and scalable in use. They enable an automatic download of EMG recordings of patients from the SmartEMG portal. The results are processed and returned in the form of.xls sheets. Scripts offer a display of data in graphs and the program’s console. Three methods of data processing were used, and each of them brought similar, anticipated results.

The analysis of processing results confirms that the Myo controller and the developed tools, constitute a system capable of giving correct information with regard to the condition of patients with tennis elbow and/or golfer’s elbow. The analysis results are satisfying because they reflect the presence of injury in a studied group of patients and also confirm that the same methodology did not show injury in a control group. There are grounds to consider the use of Myo to diagnose conditions related to enthesopathy in the humero-ulnar joint, as it is possible to determine which muscle groups are weakened.

It has been proven that the developed solutions effectively identify patients with injuries and that they record the status of their muscle activation force. It would then seem appropriate to consider using the system to monitor the rehabilitation of the described injuries over a longer period of time. This type of digital representation of the patients’ forearms condition offers more possibilities of processing and evaluation of their present and former condition than traditional methods which make use of the analog dynamometer or subjective assessment of the pain threshold.

Also, a group of six motions used in the study was determined. The “fist grip”, popular in literature, proved to be a less significant in differentiating between healthy and injured individuals. The other motions show those differences effectively. The proposed motions of pressing against the occiput and the sternum differentiated between the healthy and injured individuals at the level of index of 50.10% and 59.92% respectively. This is a good result, which confirms the applicability of these motions. Potentially, they could find their application in telemedicine, as they do not require assistance in exerting resistance during movement.

Relevant literature shows a growing interest in the Myo controller, especially in the areas of pattern classification and robotics [32–35]. Articles on the subject are concerned, among others, with the controlling of a robotic arm via the wearable Myo armband using a muscle gesture system [34]. Also, an EMG pattern classification control for an exotendon device has been proposed [35]. A paper has been written on addressing the development of a testing algorithm for pattern-recognition based strategies to control a myoelectric prosthesis [36]. At the time of writing this article, the authors had not encountered any studies regarding the application of the Myo controller in the treatment of elbow enthesopathy.
